# Role of Emerging Environmental Risk Factors in Thyroid Cancer: A Brief Review

**DOI:** 10.3390/ijerph16071185

**Published:** 2019-04-02

**Authors:** Maria Fiore, Gea Oliveri Conti, Rosario Caltabiano, Antonino Buffone, Pietro Zuccarello, Livia Cormaci, Matteo Angelo Cannizzaro, Margherita Ferrante

**Affiliations:** 1 Environmental and Food Hygiene Laboratories (LIAA), Department of Medical Sciences, Surgical and Advanced Technologies “G.F. Ingrassia”, University of Catania, 95123 Catania, Italy; mfiore@unict.it (M.F.); pietro.zuccarello@unict.it (P.Z.); marfer@unict.it (M.F.); 2 Department of Medical and Surgical Sciences and Advanced Technologies, “G.F. Ingrassia”, Section of Anatomic Pathology, 95123 Catania, Italy; rosario.caltabiano@unict.it; 3 Department of General Surgery and Specialty Medical Surgery, Endocrine surgery, A.O.U. Policlinico—Vittorio Emanuele P.O. G. Rodolico, University of Catania, 95123 Catania, Italy; abuffone@unict.it; 4 Hygiene and Preventive Medicine Specializaton School, Department of Medical and Surgical Sciences and Advanced Technologies, “G.F. Ingrassia”, 95123 Catania, Italy; livia.cormaci23@gmail.com; 5 Chirugia Generale, Department of Medical and Surgical Sciences and Advanced Technologies, “G.F. Ingrassia”, 95123 Catania, Italy; cannizzaromatteoangelo@yahoo.it

**Keywords:** thyroid, cancer, environment, risk, toxics, ECDs, carcinogenicity

## Abstract

Environmental factors are recognized as risk factors of thyroid cancer in humans. Exposure to radiation, both from nuclear weapon or fallout or medical radiation, and to some organic and inorganic chemical toxicants represent a worldwide public health issue for their proven carcinogenicity. Halogenated compounds, such as organochlorines and pesticides, are able to disrupt thyroid function. Polychlorinated biphenyls and their metabolites and polybrominated diethyl ethers bind to thyroid, transport proteins, replace thyroxin, and disrupt thyroid function as phthalates and bisphenolates do, highly mimicking thyroid hormones. A better knowledge of environmental risks represents a very important tool for cancer prevention through true risks prevention and management. This approach is very important because of the epigenetic origin’s theory of cancer. Therefore, the aim of this review was study the association between environmental agents and thyroid cancer promotion.

## 1. Introduction

Air, water and soil contamination have a detrimental effect on people’s health. Several diseases are associated to environmental pollutants including male/female fertility, cancers, metabolic and thyroid conditions, allergy, etc. [1,2,3,4,5,6,7,8,9].

Among cancers, thyroid carcinoma is the most common endocrine tumor with the largest annual incidence increase in the United States also due to the improvement of the diagnostic technologies. We forecast that thyroid cancer will be the third most common cancer in women by 2019, with a cost of $19–21 billion in the United States alone, and it will represent the fourth cancer diagnosis by 2030 [10,11]. Thyroid carcinoma occurs more frequently in females (2–4 times) than in males [12], and is rare in children and adolescents, even if malignancy of thyroid nodules occur more frequently in young individuals [11,13]. Currently, thyroid cancer is responsible for 567,000 cases worldwide. The global incidence rate in women of 10.2 per 100,000 is 3 times higher than in men; the disease represents 5.1% of the total estimated female cancer burden. Mortality rates are much lower, with rates from 0.4 to 0.5 in men and women, and an estimated 41,000 deaths. Thyroid cancer incidence rates are the highest among both men and women in the Republic of Korea. Incidence rates are higher among women than among men in North America (especially in Canada), Australia/New Zealand, as well as Eastern Asia; female rates also are high in several countries in the Pacific, including New Caledonia and French Polynesia [14]. The Italian AIRTUM Working Group reported that in adolescents (15–19 years) between 1988 and 2008, a significant increase in incidence rates was observed for all malignant tumors, in particular Hodgkin’s lymphoma and thyroid cancer (age-period-cohort: +6.1%). The increase in cancer incidence observed until the end of nineties has halted, with the sole exception of thyroid cancer in young people [13,14].

The thyroid carcinoma may originate from follicular thyroid cells or parafollicular cells.

Follicular cell-derived carcinomas can be classified into:

1. papillary thyroid cancer (PTC; 75–85% of cases) often with an excellent prognosis;
2. follicular thyroid cancer (FTC; 10–20% of cases);
3. Hürtle cell carcinomas (or oxyphilic cell carcinoma), rare and with prognosis similar to FTC;
4. poorly differentiated thyroid cancer (PDTC), an uncommon form of thyroid carcinomas accounting for less than 5% of all cases;
5. anaplastic thyroid cancer (ATC), aggressive undifferentiated tumors with a mortality near to 100% of patients, and finally.

Differentiated thyroid cancers represent about 90% of all thyroid cancers including the papillary and follicular histotypes, and have generally a favorable prognosis [11,12]. Several studies report that environmental factors, lifestyle, and comorbidities could directly contribute to this phenomenon; in particular, the environmental exposures to pollutants are of interest for epidemiologists due to their ubiquitous presence and the high dispersion in several environmental compartments including food chain. Often, no sufficient data are available about carcinogenicity and/or capacity to affect the human endocrine system for many pollutants, and because of the lack of full understanding of the pathologic phenomenon, it is difficult at present to propose an efficient prevention strategy for thyroid cancer. So, we performed a comprehensive review of the reported environmental risks significantly associated to thyroid carcinogenesis.

## 2. Material and Methods

Research was performed according to the PRISMA (Preferred Reporting Items for Systematic Reviews and Meta-analyses) guidelines [15]. The international databases PubMed, Web of Sciences, and Scopus were used to identify articles and studies that evaluated the relationship between environmental risks exposure and thyroid cancer.

In our search strategy, we applied the following combinations of keywords—environmental risk, thyroid, cancer, pesticides, Persistent Organic Pollutants (POPs), Endocrine disrupting chemicals (ECDs), Bisphenol A (BPA), phthalates, metals, radiation, and Polychlorinated Biphenyl (PCBs). We also used combinations of the keywords such as environmental risks of thyroid cancer, chemical pollutants and thyroid cancer, environmental exposures and thyroid cancer, pesticides and thyroid cancer, and POPs and thyroid cancer. Two investigators independently reviewed each publication to be included in the review, applying the study selection criteria, and then reported.

The authors concluded that the studies were of limited quantity to conduct meaningful meta-analysis of all studies. We included case–control studies, cohort studies, cross-sectional studies, interventional studies, reviews, systematic review and meta-analysis, published between 2009 and 2019. Only the articles in English and French were considered. We excluded letters, conference, abstracts or editorials; poor quality articles; in vitro and in vivo studies.

## 3. Chemical Pollutants

Environmental endocrine disrupting chemicals (EDCs), including pesticides and industrial chemicals produce deleterious effects on health. Long-term human studies about the thyroid-related outcomes of ECDs are still lacking. Exposure to ECDs is not always correlated with reductions in thyroid hormone in humans [16,17]. The lifelong chronic and sub-chronic exposure to a large mixture of chemicals and the large physiological variation in thyroid hormone levels among individuals make human studies very difficult to perform [17,18].

### 3.1. Pesticides

Organochlorine pesticides were introduced in the 1940s for their effectiveness against a variety of insects. However, like polychlorinated biphenyls (PCBs), they were also banned in developed countries during the 1970s and 1980s. Dichlorodiphenyltrichloroethane (DDT, along with its major metabolite dichlorodiphenyldichloroethylene (p,p-DDE) is only one of the many organochlorine pesticides, which was the most studied for its activity as ECD.

DDT and HCB (hexachlorobenzene) show developmental defects in thyroid hormone production [19]. DDT decreases the thyroid hormones activity, while HCB metabolizes giving highly toxic compounds, targeting thyroid hormones.

Several studies examined the relationship between pesticide exposures and some cancers including thyroid cancer, but none of the studied pesticides (alachlor, atrazine, chlorpyriphos, glyphosate, imazethapyr, and metolachlor) were associated with increased risk of cancer [19,20].

### 3.2. Phthalates and Bisphenol A

Phthalates are widely used as plasticizers to improve the plasticity, flexibility, and the malleability of the materials. Humans are exposed to phthalates through several routes such as inhalation, ingestion, and dermal exposure [21]. Despite the current ban for the use of some phthalates such as Di-(2-Ethylhexyl) phthalate (DEHP) in children’s toys in US and EU, many types of phthalates are still employed in various other industries, i.e., in cosmetics, paints, food packaging, cleaning agents, and medical devices (tablet coatings, blood bags and tubes, drugs packaging, etc.). Phthalates aren’t accumulating in the body, but they are metabolized and mainly excreted in the urine (within hours or few days) [7,21]. Phthalates have endocrine-disrupting ability, and thyroid is one of the major target organs. Phthalates are particularly interesting EDCs due to the continuous and long-term exposure of the whole world’s population. Literature shows that many phthalates (including butyl benzyl phthalate (BBP) and DEHP) are able to bind and activate the estrogen receptor and angiogenesis, and tumor progression processes through the regulation of vascular endothelium growth factor (VEGF). In fact, these compounds induced a dose-dependent increase of VEGF secretion in MELN’s cells (Melbourne cells model) with constant expression of ERα receptor (estrogen receptor alpha) [7].

Bisphenol A (BPA) is another plasticizer that interferes with thyroid hormone. BPA disturbs the synthesis and secretion of endogenous hormones in humans and other organisms, and mimic endogenous hormones, disrupting the normal function of the endocrine systems. In fact, BPA is structurally similar to 17b-estradiol, and it competes with endogenous estrogens to bind membrane estrogen receptors [22]. Some studies indicate several mechanisms by which BPA may interfere with thyroid function; in fact BPA inhibits human recombinant TPO (thrombopoietin) activity, and at receptor level, BPA binds to the thyroid hormone receptor (TR) as a weak ligand and acts as an antagonist to T3, thus inhibiting TR-mediated transcriptional activity. No human studies about the thyroid-disrupting effects of BPA have been performed, but results from animals study support the hypothesis of BPA as a risk factor for thyroid cancer. In particular, according to current fetal origin hypothesis of cancer, the fetus and infant are the most vulnerable to BPA exposure effects [23].

### 3.3. Polychlorinated Biphenyls (PCBs)

Polychlorinated biphenyls (PCBs) are a class of synthetic, lipophilic, and persistent compounds that were widely used in industrial and consumer products for decades before their production was banned in the late 1970s. PCBs bioaccumulate in the food chain due to their lipophilicity, and consumption of high size fish (tuna, shark, swordfish, etc.) is considered a primary source of human exposure. PCBs have thyroid-disrupting effects [24]. A study reviewed available epidemiologic studies, reporting discordant results among studies of correlations, and suggesting that there were no clear studies on dose–response associations [19].

### 3.4. Perfluorinated Compounds

The perfluorinated compounds (PFC) comprises, e.g., perfluorooctanoic acid (PFOA) and perfluorooctane sulfonate (PFOS) and possess surface protection properties. PFC are a broad range of compounds used in numerous applications including stain repellents for textiles, additive to paper products, and in aqueous film forming foams used to fight electrical fires. For this ability, they are used for many consumer products as stain- and oil-resistant PFCs, which are extremely persistent in the environment, so they have been added to the Stockholm list [25]. A large study carried out on 506 employees, in a PFC manufacturer company, showed negative associations between PFOA and FT4, indicating that exposure to high levels of PFOS may interfere with human thyroid function. Results of the more recent study NHANES, carried out in the US, showed that women with high levels of PFOA and men with high levels of PFOS are more likely to report current thyroid disease [25]. PFC compounds appear to interfere with thyroid hormone metabolism [16,23,25].

### 3.5. Brominated Flame-Retardants

Brominated flame retardants (BFRs) are compounds widely used in consumer products including electronics, vehicles, plastics, and textiles to reduce flammability [16].

Since the 1970s, polybrominated diphenyl ethers (PBDEs) have been widely used as flame retardants in the United States in a variety of commercial and household products, such as foam padding, textiles, electronic equipment, airplanes and automobiles, etc. [16,26]. Three formulations, PentaBDE, OctaBDE, and DecaBDE (banned at the end of 2013), were used in the United States until 2004; in fact, manufacture of PentaBDE and OctaBDE was banned because of the concerns about toxicity and the bioaccumulation of PBDEs in serum and breast milk [27]. In 2010, the US Environmental Protection Agency promulgated rules to further reduce imports of products containing these compounds [26]. Among the congeners, only the thyroid carcinogenicity of decabromodiphenyl ether (BDE-209), the main congener in the DecaBDE formulation, has been evaluated in rats; no human study was available with the exception of Aschebrook-Kilfoy et al. [10]. Despite the demonstrated disruption of thyroid homeostasis by environmental levels of PBDEs, the modest number of cases does not provide support for an increased risk of thyroid cancer [10]. Thirty-six epidemiological studies were reviewed by Kim and colleagues about possible outcomes associated with BFR exposure including effects and alteration on thyroid function. Although there is significant evidence on the health effect of exposure to BFRs, further epidemiological investigations, particularly, among children are needed [28].

### 3.6. Perchlorates

Perchlorate has well-known antithyroidal effects, and for these characteristics, it has been used earlier in diagnosis and treatment of thyrotoxicosis. It is used in the production of ordnance and fireworks, and the presence of the perchlorate on drinking water in the US was a source for concern in the past years. Consumption of food and water containing perchlorates are the most relevant routes of exposure for the general population [29]. Despite the well-described antithyroid effects of perchlorate, the lowest effective level from environmental contamination levels still remains unclear. It remains controversial whether environmentally occurring levels of perchlorate ions have any effects on humans, and especially neonatal, thyroid function, as the results from human observational studies are conflicting [16,23].

### 3.7. Metals

Metals present in air, water, and food may impact human health [30,31,32,33,34,35,36,37,38]. Some metals are essential (chromium, iron, copper, magnesium, and zinc), but at high concentration may be toxic, while others metals do not play any specific role in biological processes (aluminum, nickel, cadmium, mercury, and lead).

Heavy metals are byproducts of incinerators, combustion of gasoline or diesel fuel as elemental components of PM_10_, PM_2.5_ (cars, trucks, airplanes), smelters, paints, insecticides, and agriculture products such as disinfectants; they can be absorbed by inhalation, ingestion [39,40,41,42], or even skin contact.

Several metals have been classified as definite or probable carcinogens by the International Agency for Research on Cancer (IARC); arsenic, beryllium, cadmium, chromium, and nickel are carcinogenic. Especially cadmium, mercury, arsenic, lead, manganese, and zinc show EDCs capacity perturbing the hormonal system, and as carcinogens, promoting malignant transformation [43]. The effect of metals on the human thyroid is still poorly studied; in fact, for some heavy metals, no data are available currently [44]. Similar to other carcinogens, the transforming effect of heavy metals is higher in developing organisms, such as the fetus (through the mother) and individuals in early childhood [45].

Among these, cadmium is considered a category I carcinogen, and it is able to accumulate in liver, pancreas, kidneys, and also in the thyroid. Cadmium in blood correlates with its concentrations in the thyroid. In chronic cadmium toxicity, multinodular goiter, thyroglobulin hyposecretion, and parafollicular cell hyperplasia are frequent [45].

The capacity of cadmium to bioaccumulate and its persistence in the environment for many years increases the associated health hazards. Epidemiological and experimental data prove that cadmium can disrupt thyroid gland function even at very low environmental doses, both at target gland and extra gland level. The role of cadmium in autoimmune disease and in thyroid cancer has been demonstrated [46].

A Korean study showed that high levels of cadmium in thyroid tissue are associated with more advanced stage of thyroid cancer in women. Considering that traditional Korean dietary pattern is related with high risk for cadmium exposure, this result is an important information concerned with thyroid cancer [47].

Several metabolic pathway are supported by manganese (Mn)-containing enzymes. For the general population, the major source of exposure to manganese is dietary, although drinking water may constitute an additional source in some regions. Environmental or occupational exposure to high levels of Mn causes a neuropathy similar to idiopathic Parkinson’s disease, known as manganism. Dopamine and its metabolites are altered in manganism and in Parkinson’s disease; moreover, dopamine and dopaminergic receptors are involved in neurodevelopment and TSH (thyroid stimulating hormone) modulation. This suggests that excessive Mn dose exposure during gestation is linked to pathological neurodevelopmental outcomes due to altered thyroid hormones’ levels. Experimental evidence about quantitative increase in the incidence of thyroid tumors in exposed mice to Mn does not provide any clear evidence; in addition, the available occupational and environmental epidemiological evidence is still equivocal [48].

Lead (Pb) is a metal with controversies about its carcinogenicity. Li and colleagues conducted a study to evaluate the correlation between Pb and thyroid functions in Chinese patients with different thyroid diseases (96 PTC, 10 nodular goiter (NG), and 7 thyroid adenoma (TA)). Serum triiodothyronine (T3), free triiodothyronine (FT3), free thyroxin (FT4), thyroid stimulating hormone (TSH), and serum lead were evaluated with appropriate methodologies. Compared to PTC, the level of lead was significantly higher in TA, and lower in NG (*p* < 0.05). This difference remained significant in females when stratified by sex. Serum lead was negatively correlated with TSH (*r_s_* =  −0.27, *p* < 0.05) in PTC group. T3 was positively related to lead at quartile (*r_s_* = 0.61, *p* < 0.05) in PTC group. No significant correlations were observed between lead and FT3 or FT4 in any group. The results of this study suggested that lead might have different etiological roles in these three thyroid diseases including thyroid cancer [49].

Vanadium (V) is a metal existing in different oxidation states and the most common form is the vanadiumpentoxide (V_2_O_5_). All vanadium compounds are toxic. A carcinogenic role of vanadium on the thyroid was recently proposed by some authors. However, no studies have evaluated the risk in humans and/or animals of thyroid disease after exposure to vanadium. Fallahi et al. studied this new hypothesis evaluating the effect of V_2_O_5_ on proliferation, and chemokine secretion in normal thyrocytes. Through its study, the authors demonstrated that V_2_O_5_ promotes the induction and perpetuation of an inflammatory reaction in the thyroid through the induction of the secretion of T-helper (Th)1 chemokines into the thyroid and synergistically increasing the effect of important Th1 cytokines such as interferon (IFN)γ and tumor necrosis factor (TNF)α [50].

### 3.8. Metalloids

Micronutrients, mostly iodine (I) and selenium (Se), are required for thyroid hormone synthesis and function [3,51]. It has been shown that iodine and selenium have an important role in thyroid autoimmunity [45]. Iodine prophylaxis reduce iodine deficiency-related to thyroid disorders. In the Danish population, a 53% higher incidence of spontaneous overt hypothyroidism (probably autoimmune) was observed in mild iodine deficiency. Instead, iodine excess is associated with the onset of autoimmune thyroiditis [52]. In differentiated thyroid cancer (DTC), radioiodine ^131^I is administered in medical diagnostic and treatment procedures to eliminate residual normal thyroid tissue after thyroidectomy, for more details see Section 4.1.

Selenium is a trace mineral essential to human health and, in the form of selenoprotein, performs various functions in healthy status with a normal metabolism [53]. Selenium whose necessary intake ranges from 60 to 75 μg/day, exerts other several effects including immunological responses, homeostasis, cell growth, and viral defense. Moreover, it is necessary for normal thyroid function in humans because of its presence as a component in several enzymes such as glutathione peroxidases, deiodinases, and thioredoxin reductases to participate actively in the protection against free radicals and oxidative damages [3,30,45,54]. Selenium is involved in the development of thyroid cancer (TC) [52,54], but its role in cancer treatment is still debated [3,55,56]; in fact, a high selenium intake may have unfavorable effects on the endocrine system and particularly on the thyroid status [3,45,52].

In a Chinese cross-sectional study, carried out in two counties of Shaanxi Province, the relationship between selenium status, dietary factors, and pathological thyroid conditions were investigated. In the adequate-selenium concentration, the prevalence of pathological thyroid disorders (subclinical hypothyroidism, hypothyroidism, AT, and enlarged thyroid) was significantly lower than that in the low-selenium category (18.0 versus 30.5%; *p* < 0.001) [57]. Elevated circulating selenium level was associated with reduced odds ratio (95% confidence interval) of subclinical hypothyroidism (0.68; 0.58, 0.93), hypothyroidism (0.75; 0.63, 0.90), autoimmune thyroiditis (AT) (0.47; 0.35, 0.65), and enlarged thyroid (0.75; 0.59, 0.97) [57].

Wu et al.’s paper [57] evaluated the real effectiveness of selenium supplementation in Hashimoto’s thyroidites, measuring thyroid-stimulating hormone (TSH), thyroid hormones, TPOAb and thyroglobulin antibodies (TgAb) levels, and thyroid echogenicity after 6 months of l-selenomethionine treatment. Authors showed that the short-term l-selenomethionine supplementation has a restricted impact on the natural course in euthyroid HT [45,58].

### 3.9. Nitrates

Nitrates are widespread contaminants. Excessive nitrates uptake via oral route increases nitrites production, which leads to the development of hypoxia in the blood, especially in children, and to the overproduction of nitric oxide (NO), which is a recognized carcinogenic and it is involved in other different pathogenic mechanisms [59,60,61].

Nitrate intake from drinking water and dietary sources can interfere with the uptake of iodide by the thyroid, so this contaminant represents a risk for thyroid functions [62]. Nitrate causes the reduction of production of thyroid hormones that result in a compensatory increase in thyroid stimulating hormone (TSH), a sensitive indicator of thyroid function [63]. A high TSH release has been shown in animals to induce hypertrophy and thyroid disease including carcinoma; however, self-reported hypothyroidism and hyperthyroidism among a cohort of post-menopausal women in Iowa was not associated with average nitrate concentrations in public water supplies [62].

Xie et al.’s meta-analysis showed that consumption of food rich in nitrate wasn’t significantly associated with gastric cancer risk (risk ratio or RR = 1.24, 95% CI = 0.89–1.72). Instead, individuals with higher nitrites consumption had an increased thyroid cancer risk when compared to the lower exposure (RR = 1.52, 95% CI = 1.12–2.05) [64].

Inoue-Choi et al. reported that among women with >10 years of exposure through public water supply with levels exceeding 5 mg/L NO_3_-N for 5 years or more, thyroid cancer risk was 2.6 times higher than that in women whose supplies never exceeded 5 mg/L NO_3_-N [65].

Nitrate concentrations, in private wells among a cohort of old order Amish in Pennsylvania (USA), were associated with increased prevalence of subclinical hypothyroidism as determined by thyroid stimulating hormone measurements, among women but not men [63].

Also therapeutic irradiation (^131^I) increases NO levels in salivary gland tissue of patients. NO is an intrinsic radiosensitizer as proved by several evidences; in fact, administration of an NO synthesis inhibitor ameliorate the dysfunction of irradiated salivary glands.

The NO-dependent effects include the promotion of genomic instability and the accumulation of DNA reduplication errors in bystander cells (unirradiated cells exhibit irradiated effects as a result of signals received from nearby irradiated cells) without the direct DNA damage seen in irradiated cells. The hydrophobic properties of NO permit its diffusion through the cytoplasm and plasma membranes, allowing this signaling molecule to easily spread from irradiated cells to bystander cells without the involvement of gap-junctional intercellular communication [66].

### 3.10. Air Pollution

WHO, through its IARC agency managed a revision on the classification of cancer-causing agents, and urban air pollution is considered a worldwide recognized cancer risk factor [67].

Particulate Matter (PM) is a proxy indicator for air pollution. It affects more people than any other pollutant. The major components of PM are sulfate, nitrates, ammonia, sodium chloride, black carbon, mineral dust, and water. It consists of a complex mixture of solid and liquid particles of organic and inorganic substances suspended in the air. Particles with a diameter of 10 microns or less (≤PM_10_) can penetrate and lodge deep inside the lungs. Even more health-damaging particles are those with a diameter of 2.5 microns or less (≤PM_2.5_) that can penetrate the lung barrier and enter the blood system. Chronic exposure to such particles contributes to the risk of developing cardiovascular and respiratory diseases, as well as of lung cancer.

About 80% of European citizens are exposed to higher concentrations of fine particulates (PM_2.5_ and PM_10_) reported as carcinogens for humans (International Agency for Cancer Research, IARC position 2015) [21].

A large prospective study showed that outdoor air pollution (PM_2.5_, NO_2_, and O_3_) wasn’t associated with death from most non-lung cancers, but positive associations were revealed with colorectal, kidney, and bladder cancer death [68]. Cong (2018) investigated the effects of outdoor air pollution from waste gas emission on multiple cancer incidences in a retrospective population-based study in Shanghai, China. Cong enrolled and reviewed more than 550,000 new cancer patients, and its results suggested that ambient air pollution from waste gas emissions was positively associated with multiple cancer incidences including thyroid cancer [69].

## 4. Physical Factors

### 4.1. External Anthropogenic Radiation

^131^I is today associated with nuclear energy, medical diagnostic, and treatment procedures. It was a significant contributor to the health hazards from open-air atomic bomb testing in the 1950s, and from the Chernobyl disaster, as well as being a large fraction of the contamination hazard in the first weeks in the Fukushima nuclear crisis. This is because ^131^I is a major fission product of uranium and plutonium, comprising nearly 3% of the total products of fission [70].

From the therapeutic point of view in differentiated thyroid cancer (DTC), radioiodine ^131^I is administered to:

- eliminate residual normal thyroid tissue after thyroidectomy;
- treat residual microscopic disease (adjuvant treatment);
- treat macroscopic or metastatic disease [71].

Treatment with ^131^I is based on the ability of tumoral thyroid tissue to incorporate iodine from the blood by the sodium/iodine transporter membrane. Expression of this transporter is lower in tumoral tissue compared to healthy tissue, and thus, the uptake of ^131^I may be reduced. Nonetheless, the beta radiation of ^131^I leads to oxidative species formation such as free radicals at an intracellular level, DNA lesion, and finally cell death, which is the main objective of the treatment [71]. However, treatment of DTC with ^131^I is still an open issue because recommendations of experts are based only on data interpretation of observational retrospective studies. The results of the prospective trials are pending, and the use of ^131^I is justified in high-risk, intermediate-risk, and low-risk patients [71,72] according to the guidelines of the American and British Thyroid Association, European and American Societies of Nuclear Medicine, the European Consensus Group, and the latest edition of National Comprehensive Cancer Network (NCCN) [71,73].

Exposure to ^131^I radioisotope in childhood is associated with an increased risk of thyroid cancer. Both iodine deficiency and iodine supplementation modify this risk. So a stable iodine supplementation in iodine-deficient populations may substantially reduce the risk of thyroid cancer related to radioactive iodine in case if its exposure in childhood following radiation accidents or during the medical diagnostic and therapeutic procedures [74].

The thyroid gland is particularly susceptible to radioiodine exposure, although thyroid tumors can also be induced by other ionizing radiation, such as alpha particles from ^238^Pu [75].

Radiation exposure from US atomic bombings (1945) impacted Japanese children’s thyroids, in particular adolescents showed an increased risk of developing thyroid cancer in the five decades after atomic radiation exposure.

The Chernobyl accident (1986) had caused the largest uncontrolled radioactive release into the environment ever recorded for any civilian activity. The ^131^I provided significant contributions to the radiation dose in the whole of Belarus and Ukraine. The contamination of fresh milk with ^131^I and the lack of prompt countermeasures resulted in high thyroid doses, particularly among children. In Chernobyl, there was an increased incidence of thyroid cancer among exposed children, and thyroid peroxidase antibodies’ prevalence was higher in radiation-exposed children (6.4% versus 2.4% in unexposed) and in adolescents exposed to radioactive fallout 13–15 years after the Chernobyl accident. Instead, no increased risk was found among adult Chernobyl liquidators, and this fact makes ambiguous the evidence of risk among exposed adults [45,70].

Nagataki and Takamura described the radiation effects on the thyroid of the more recent nuclear accident at the Fukushima Daiichi Nuclear Power Station operated by Tokyo Electric Power Company, which occurred in March, 2011. The amount of radioiodine released to the environment following the Fukushima accident was 120 Peta Becquerel. The doses released from the accident in Fukushima were lower compared to those in Chernobyl accident that increased the thyroid cancer risk. Residents near the Fukushima nuclear plant were evacuated within a few days; therefore, thyroid radiation doses were less than 100 mSv (intervention levels for stable iodine administration) in the majority of children, including less than 1 year olds. To date, screening of more than 280,000 children has resulted in the diagnosis of thyroid cancer in 90 children (approximate incidence, 313 per million) [76,77]. However, 26 experts from Japan and international organizations, including the World Health Organization (WHO), United Nations Scientific Committee on the Effects of Atomic Radiation (UNSCEAR), International Commission on Radiological Protection (ICRP), IAEA, and International Agency for Research on Cancer (IARC), reviewed and scrutinized the findings on thyroid cancer in Fukushima, highlighting that the high detection rate of thyroid cancer and benign abnormalities resulted not only from the use of highly sensitive ultrasound instruments but also from a sophisticated protocol of examination applied in the thyroid ultrasound examination, and then not attributable to exposure to radiation [77]. In fact, thyroid cancers were detected in children in their late teenage years, but no cases were found in the most vulnerable group of very young children, which further suggests that the increase is due to screening rather than radiation exposure. Any cancer needs a period of time from the genotoxic insult to be detected and also sensitive techniques such as the thyroid ultrasound examination [77].

Moreover, the literature shows no increased risk of thyroid cancer associated with living near nuclear plants [78,79]. Results of a recent meta-analysis showed a similar finding. However, there was substantial heterogeneity among incidence studies, and the results need to be interpreted cautiously. Probably, radioactive discharges from modern nuclear plants might be too small to be statistically detectable [79].

### 4.2. External and Internal Natural Radiation

Radon (Rn) is a colorless, odorless, and tasteless natural gas produced by the radioactive decay of uranium, which is found in rocks and soil. Radon-222 (^222^Rn) is a member of the radioactive decay chain of uranium-238 (^238^U). Radon-220 (^220^Rn) is formed in the decay chain of thorium-232 (^232^Th). As a part of normal radioactive decay, radon produces short-lived radioisotopes that can be inhaled and drank by humans [80]. In fact, in many countries, drinking water is obtained from groundwater sources such as springs and wells. These sources of water normally have higher concentrations of radon than surface water from reservoirs, rivers, or lakes [9]. Exposure to radon >4 picocuries per liter (pCi/L) is considered dangerous to humans due to its recognized association with lung cancer. Radon is identified as a group I carcinogen, and is the second leading cause of lung cancer in the world after tobacco smoke [75]. In a study carried out in Sicily (Italy), residents of the volcanic region of Catania province seem to have higher incidence of thyroid cancer than other populations, and it is reported that the concentration of Rn is elevated in this area; however, the authors were not able to conclude on the association of Rn exposure with the risk of thyroid cancer [81].

An ecological study across three different States in the US (Iowa, New Jersey, and Wisconsin) was carried out lately, but results didn’t find any association between elevated radon and thyroid cancer incidence in women. In fact, New Jersey had the most female thyroid cases reported (*N* = 16,906), followed by Wisconsin (*N* = 7250), and Iowa (*N* = 4236) in the period 1990–2013. Pearson correlation coefficients between radon over 4 pCi/L and age-adjusted thyroid cancer incidence rate were calculated for each of the three states, and all correlations showed no association. Also another study carried out in Pennsylvania showed no association between cumulative radon levels and thyroid cancer incidence [82].

## 5. Live in Volcanic Environment

The possibility of an association between increased incidence of thyroid cancer and active volcanoes was first introduced in 1981. Thus, the volcanic environment is a good model to investigate the possible determinants favoring thyroid cancer. Several authors noted that thyroid cancer incidences in the volcanic islands of Iceland and Hawaii were much higher than those in other countries. Very high thyroid cancer incidence was also reported in other volcanic areas as Vanuatu and in French Polynesia, and an incidence 10-fold higher was reported in New Caledonia (Australia) [44]. In the volcanic area of Mt. Etna in Sicily (Italy), as well as in other volcanic areas, a non-anthropogenic pollution with heavy metals has been documented because of frequent eruptions with gas, ash, and lava emissions polluting soil, water, and atmosphere. In addition, non-anthropogenic pollution with heavy metals, through the food chain, has biocontaminated the residents as documented by statistically significantly high metals levels in the urines and the scalp hair, compared to individuals living in adjacent non-volcanic areas [83]. The mechanism by which a volcanic environment could promote thyroid cancer is unknown. The area surrounding an active volcano can be polluted with various inorganic elements in the soil (because of lava flow and ash emission fall-out), the atmosphere (because of emitted gases and small particulates), and the water (because of groundwater source contact with gases and chemicals originating from the magma chamber deep below the surface). The soil, atmosphere, and water are all potential vehicles for incorporating volcano-originated elements into locally grown vegetables and animals with consequent bio-contamination of the volcanic area residents [83]. Individuals inhabiting a volcanically active environment more frequently have DNA damage than residents of an adjacent control area [84]. One or more of these contaminants could exert carcinogenic effects on the thyroid. The specific effect of different metals, at different concentrations, in different human tissues is mostly unknown but the hormesis effect, a biphasic dose-response relationship whose occurrence has been documented in vitro for different metals, including As, Cd, Cu, and Hg. This phenomenon is characterized by stimulation of biological effects at lower concentrations (low mM or even nM) and inhibition at higher concentrations [83]. The mechanism of hormesis is not well understood, but it’s known that it has a relevant toxicological implication on the potential impact of chronic exposure to very low levels of many toxicants [83].

## 6. Fetal Origin of Endocrine Dysfunction by Environmental Exposures and Epigenetics Evidences

Even a small reduction of environmental pollutants could confer significant population health benefits. The fetal origin of endocrine dysfunction in the adult is a new hypothesis and it refers to the exposure to an environmental chemical during development that may affect the physiology of the offspring later in life [85,86,87,88]. In fact, humans’ maternal exposure to EDCs is the first source of fetal exposure from temporal point of view, and some ECDs has been identified already in amniotic fluid [22,88,89], umbilical cord blood [22], and other body fluids [22,90]. This exposure continues after birth through breast feeding [91,92,93], infant foods [93,94,95], and direct contact to the environment. These abundant man-made sources of EDCs result, for e.g., for phthalates, in human exposure was estimated at 1.7–52.1 µg/kg/day [88], and under specific circumstances, infants can be exposed to up to 3 times as much [88].

EDCs not only involve genetics but also epigenetic mechanisms in tumors’ generation. In humans, there is now growing evidence that ECDs such as PCBs, BPA, and phthalates have thyroid-disrupting effects. Yoshinaga study showed that exposure to hydroxylated-PCBs at environmental levels during the first trimester of pregnancy can affect neonatal thyroid hormone status [96]. It has also been shown that early exposure to certain environmental chemicals with endocrine-disruption activity as pesticides may interfere with neonatal thyroid hormone status [97] representing a risk for thyroid tumor in the adult age.

Chromosomal rearrangements induced by chemical and therapeutical causes contribute to the majority of oncogenic fusions found in cancer. DNA fragile sites are sensitive to a variety of chemicals and have been identified in regions with deletions and chromosomal rearrangements.

Epigenetic mechanisms, especially aberrant methylation of DNA and microRNAs, are likely to play an important role in thyroid tumorigenesis [95]. Well-studied epigenetic modifications that affect gene expression also include the post-translational modification of histone proteins (by acetylation, methylation, phosphorylation, ubiquitylation, sumoylation, proline isomerization, and adenosine diphosphate-ribosylation) [98,99].

The rearrangements result in the disruption of genetic material, which can cause the expression of oncogenic fusion proteins or the abnormality or, finally, the gene silencing often reported as the main process involved in tumor suppression [99].

In the development of PTC e.g., several genetic factors are involved such as the activation of the mitogen-activated protein kinase (MAPK) signaling pathway resulting by point mutations within BRAF gene (40% of PTC cases) or RAS (15% of PTC cases, exclusively the follicular variant) or RET/PTC (REarranged during Transfection-RET) rearrangement (18% of PTC cases).

Especially for RET/PTC rearrangements in genes, some environmental agents such as benzene (in cigarette smoke and automobile exhaust), diethylnitrosamine (DEN) (in cigarette smoke, pesticides, and treated meat), and chemotherapeutic drugs can significantly increase fragile site breakage, and so these pollutants are positively associated with the risk of thyroid cancer [100,101].

## 7. Conclusions

This review described the mechanisms that determine an increased risk of thyroid cancer as a consequence of environmental risk factors (Table 1). Many groups of chemicals may be hazardous and negatively affect thyroid as showed by experimental studies. However, only the effects of environmental levels of PCBs have been extensively investigated in humans through epidemiological studies. Most chemicals have been studied sporadically, and research results are not always consistent about the thyroid cancer risk. There is substantial evidence that PCBs have adverse effects on thyroid function, and other halogenated compounds, BPA, some metals, metalloids, and phthalates suggest that these chemicals also have thyroid-disrupting properties, representing a risk factor for the tumor promotion.

However, based on our review supported by the published data, we can hypothesize that thyroid cancer may derive from the simultaneous coexistence of some conditions such as:

(1) excessive nitrate uptake via drinking water that increases nitrite production and so, leads to the development of hypoxia in the blood, especially in children, and to overproduction of NO, which is a carcinogenic compound;
(2) natural radiation exposure but also therapeutic radiation exposure of the salivary glands, e.g., by dental X-ray examination, may lead to an increased plasma levels of NO;
(3) if one or both of these processes coincide with radiation exposure of the thyroid, the considerably increased NO concentrations in the body presumably enhance the carcinogenic effect of the radiation;
(4) iodine and/or selenium deficiency;
(5) air pollution exposure and live in environment at risk such as volcanic areas.

The unavoidable lifelong human exposure to mixtures of such environmental chemicals raises serious concerns about their carcinogen potential versus thyroid especially if this occurs during sensitive developmental periods. Pregnant women and their fetuses, premature children, infants, and toddlers are particularly sensitive to permanent effects on thyroid development, function, and induction of thyroid cancer.

High efforts will be necessary to avoid overdiagnosis and overtreatment, which may carry its own health risks in people.

Future studies are needed to investigate the levels of exposure to persistent organic pollutants (POPs), and control for potential confounders such as occupational and lifestyle factors, or exposure to multiple substances with the potential to cause thyroid cancer.

Additional studies are necessary in order to improve the knowledge on the mechanism causing thyroid cancer, and especially, identify the specific environmental elements that pose a risk of the disease incidence in humans.

## Figures and Tables

**Table 1:** Environmental factors and thyroid cancer risk.

Risk Factor	Sources	Mechanism of Action	References
**Chemical Pollutants**			
PCBs	Found in coolants and lubricants, multiple cogeners, lipophilic	TR agonist/antagonist, can alter levels of T4 and TSH, thyroid-disrupting effects	[19,24]
Pesticides	Used as pesticide on crops	Induce glucuronidate T4, accelerating metabolism	[19,20]
PFCs	Used as stain repellents for textiles, additive to paper products, and in aqueous film forming foams used to fight electrical fires	Interfere with thyroid hormone metabolism	[16,23,25]
BFRs	Used in consumer products including electronics, vehicles, plastics and textiles to reduce flammability	Bind to TRs, displaces T4 from binding proteins, disrupt the thyroid homeostasis	[10,16,26,28]
BPA	Used in plastic bottles, CDs, DVDs, thermal paper	Antagonize TR. It interferes with the synthesis and secretion of endogenous hormones binds to the TR and acts as an antagonist to T3	[22,23]
Phtalates	Used in cosmetics, paints, food packaging, cleaning agents and medical devices	Induction of a dose-dependent increase of VEGF secretion in MELN’s cells with constant expression of ERα receptor	[7,21]
Perchlorates	Rocket fuel, fertilizer, smoking, production of ordnance and fireworks	Inhibits iodine uptake, exert antithyroid effects	[16,23,29]
Metals (Cd, Mn, Pb, V)	Byproducts of incinerators, combustion of gasoline or diesel fuel, elemental components of PM_10_, PM_2.5_ (cars, trucks, airplanes), smelters, paints, insecticides, and agriculture products such as disinfectant, soil erosion	Induction of inflammation and immune response to autoantigens; production of reactive oxygen species such as NO Induction of inflammatory reaction in the thyroid through the induction of the secretion of T-helper (Th)1 chemokines into the thyroid and increase the effect of important Th1 cytokines such as (IFN)γ and (TNF)α	[30,36,39,42,43,44,45,46,47,48,49,50]
Metalloids (I, Se)	Byproduct in the refining of these ores, glassmaking, pigments	Participate actively in protection against free radicals and oxidative damages	[3,30,45,51,52,53,54,55,56,57,58]
Nitrates	Fertilizers	Overproduction of cellular NO, genomic instability, thyroid hypertrophy	[59,60,61,62,63,64,65,66]
**Physical factors**			
^131^I	Radioactive discharges, atomic bombings, cancer therapy	Oxidative species formation at an intracellular level, DNA lesion, cell death	[45,70,71,72,73,74,75,76,77,78,79]
Radon	Byproduct of natural radioactive decay of uranium and thorium	Oxidative species formation, DNA lesion	[75,80,81,82]
Air pollution (PM)	Industry, natural fires, urban traffic, etc.	Induction of inflammation and immune response to autoantigens; production of reactive oxygen species such as NO	[67,68,69]
Live in volcanic area	N.A.	DNA damage, hormesis effects	[44,83,84]

PCBs, polychlorinated biphenyls; TR, thyroid hormone receptor; T4, thyroxine; TSH, thyrotropin; PBDE, polybrominated diphenylethers; HT, Hashimoto’s thyroitidits; BPA, Bisphenol-A; TPO, thyroid peroxidase; n.a., not applicable; NO, Nitrogen oxide; PFCs, perfluorinated compounds; BFRs, Brominated flame retardants; (Th)1, T-helper chemokines; (IFN)γ, interferon factor and (TNF)α, tumor necrosis; Cd, cadmium; Mn, manganese; Pb, lead; V, vanadium; I, iodine; Se, selenium.
